# Soluble Soybean Polysaccharide Improves Quality and Shelf Life of Peanut Butter

**DOI:** 10.3390/foods14132180

**Published:** 2025-06-22

**Authors:** Liangchen Zhang, Liyou Zheng, Jian Sun, Sameh A. Korma, Fahad Al-Asmari, Mengxi Xie, Miao Yu

**Affiliations:** 1 Institute of Food and Processing, Liaoning Academy of Agricultural Sciences, Shenyang 110161, China; 2 School of Biological and Food Engineering, Anhui Polytechnic University, Wuhu 241000, China; zhengliyou@ahpu.edu.cn; 3 Department of Food Science, Shenyang Agricultural University, Shenyang 110866, China; 4 Department of Food Science, Faculty of Agriculture, Zagazig University, Zagazig 44519, Egypt; sameh.hosny@zu.edu.eg; 5 School of Food Science and Engineering, South China University of Technology, Guangzhou 510641, China; 6 Department of Food Science and Nutrition, College of Agriculture and Food Sciences, King Faisal University, P.O. Box 400, Al-Ahsa 31982, Saudi Arabia; falasmari@kfu.edu.sa

**Keywords:** peanut butter, soluble soy polysaccharides, stability, quality, shelf life, oil separation

## Abstract

Peanut butter, a plant-based spread, has gained global prominence due to the increasing consumer demand for nutritious convenience foods and the rising adoption of plant-based diets. However, oil separation during storage and transportation accelerates the oxidative rancidity and reduces the shelf life of peanut butter. Enhancing peanut butter stability by minimizing oil separation is therefore essential. This study investigates the effect of soluble soybean polysaccharides (SSPSs) on the quality and shelf life of peanut butter. Optimal processing conditions were established by adding 1.7% SSPS (*w*/*w*), heating the mixture to 85 °C for 40 min, and then cooling it to 1 °C. The addition of SSPSs significantly increased the lightness of the peanut butter without altering its red-green color characteristics. Furthermore, SSPS incorporation improved its textural properties by increasing hardness and cohesiveness. Nutritional analysis showed that SSPS supplementation elevated proximate composition parameters (moisture, ash, carbohydrates, and fiber) while slightly reducing acid and peroxide values. Scanning electron microscopy revealed that SSPSs enhanced the internal network structure of peanut butter, inhibited oil migration, and reduced centrifugal emulsification rates. First-order kinetic models based on acid and peroxide values were developed to predict the effects of SSPSs on shelf life. Both the model predictions and experimental data confirmed that SSPS addition effectively extends the shelf life of peanut butter.

## 1. Introduction

Peanut butter is one of the most widely consumed and important plant-based spreads, valued for its creamy texture, distinctive roasted peanut flavor, and high protein content (approximately 27%) [1,2]. Due to its high fat content (approximately 50%) [2,3], peanut butter is prone to oil separation during storage and transportation. This separation promotes oxidative rancidity, which shortens shelf life, produces off-flavors, and deteriorates the nutritional value, texture, and overall safety of peanut butter [1,4,5]. To address this issue, several strategies have been employed, including raw material selection [6], improvements in processing technologies [7], and the incorporation of emulsion stabilizers [4,8,9,10]. Among these, the use of emulsion stabilizers is considered one of the most effective, convenient, and cost-efficient approaches to controlling oil separation in peanut butter.

Rice bran wax has been added to improv the stability of peanut butter [10]. In addition, greater than 1% additions of freeze-dried hydroxypropyl methylcellulose and methylcellulose as stabilizers into peanut butter could allow it to maintain a stable period of more than 6 months [11]. However, there is a common problem in that the sensory quality of peanut butter decreases after adding stabilizers. Therefore, it is particularly important to find an additive that can improve the oil stability of peanut butter without reducing its sensory quality. Soluble soybean polysaccharide (SSPS) is a water-soluble dietary fiber extracted from soybeans, primarily from the cotyledons [12,13]. SSPS possesses excellent water-holding capacity, low viscosity, and high stability in aqueous solutions, even under heated or acidic conditions [14]. In addition to its functional properties, dietary fiber intake has been associated with numerous health benefits, including a reduced risk of cardiovascular disease, obesity, and certain cancers [15]. SSPS is widely used in the food industry not only for its nutritional value but also for its functional roles in dispersion [16], stabilization [17], emulsification [18], adhesion [19], and film formation [20] as a food additive [21].

The present study aims to evaluate the effect of SSPS incorporation on the quality and shelf life of peanut butter. Initially, the optimal SSPS concentration was determined using a single-factor experimental design to improve the centrifugal emulsification efficiency of peanut butter. Subsequently, the effects of SSPS on its nutritional composition and sensory properties were assessed by analyzing texture, color, proximate nutrient content, and microstructure. Furthermore, based on the first-order kinetic model of oil rancidity oxidation, acid value and peroxide value kinetic models were established to predict the impact of SSPSs on shelf life. The model predictions were validated through experimental verification to assess the effectiveness of SSPS in extending the shelf life of peanut butter.

## 2. Materials and Methods

### 2.1. Peanut Seeds and Chemicals

Two peanut cultivars, Baisha and G965, were obtained from the Liaoning Peanut Research Institute (Fuxin, China). Food-grade SSPSs were purchased from Zhejiang YINO Biotechnology Co., Ltd. (Lanxi, China). Food-grade sucrose and sodium chloride (NaCl) were sourced from a commercial supplier. Chromatography-grade solvents, including *n*-hexane and methanol, were obtained from Merck (Rahway, NJ, USA). Analytical-grade reagents sulfuric acid, anhydrous ethanol, potassium hydroxide, ascorbic acid, anhydrous ethyl ether, petroleum ether, potassium iodide, phenolphthalein, and sodium thiosulfate were supplied by the Sinopharm Chemical Reagent Group Co., Ltd. (Shanghai, China).

### 2.2. Optimization of Peanut Butter Processing with SSPSs

#### 2.2.1. Preparation Process

**Figure 1:**
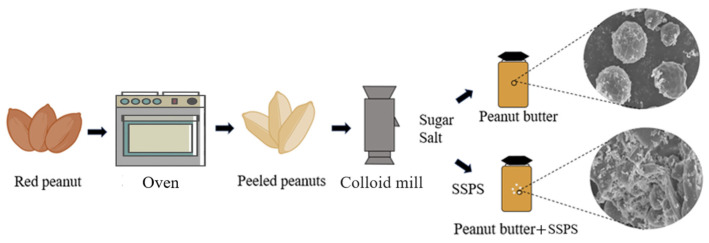
Peanut butter and SSPS peanut butter preparation diagram.

Peanut butter was prepared using a modified version of the method [6]. Only shelled, mature, and undamaged peanut kernels free from mold or physical defects were selected for processing. The kernels were evenly spread in a single layer on a stainless-steel tray (530 × 320 mm) and roasted at 160 °C for 45 min using an oven (model BS5053W, Guangdong Midea Kitchen Appliance Manufacturing Co., Ltd., Foshan, China). After roasting, the peanuts were cooled to room temperature and manually deshelled to remove their skins. The peeled peanuts were then mixed with 1.0% sodium chloride and 4.0% sucrose (*w*/*w*) before grinding. The mixture was ground for 3 min using a commercial grinder (Xingshi Machine Factory, Guangdong, China) to produce peanut butter. SSPS was subsequently added to the peanut butter and mixed thoroughly. The mixture was stirred continuously at 80 °C for 30 min to ensure the uniform incorporation of the SSPS. After mixing, the peanut butter was transferred into sterilized glass jars and allowed to cool at room temperature. Then, the jars were stored at 4 °C until further analysis (Figure 1).

#### 2.2.2. Determination of the Centrifugal Emulsification Rate of Peanut Butter

The centrifugal emulsification rate of peanut butter was measured based on previously reported methods [22,23], with minor modifications. Briefly, a known quantity of peanut butter was placed into a 50 mL centrifuge tube, and the total weight (sample + tube) was recorded as M2, while the weight of the empty tube was denoted as M1. The samples were centrifuged at 5000 rpm for 30 min at 20 °C using a centrifuge (TGL-15B, Shanghai Anting Scientific Instrument Factory, Shanghai, China). After centrifugation, the tubes were inverted and left undisturbed for 30 min to allow complete oil separation. The separated oil was carefully removed, and the weight of the remaining sample and tube was recorded as M3.

The centrifugal emulsification rate (%) was calculated using the following formula:

Centrifugal emulsification rate (%) = (M2 − M3)/(M2 − M1) × 100(1)

For reference, the centrifugal emulsification rate of peanut butter without SSPS was measured to be 12.23%.

#### 2.2.3. Single-Factor Experimental Design for Peanut Butter Processing with SSPS

SSPS was incorporated into 500 g of natural peanut butter at final concentrations of 0.5, 1.0, 1.5, 2.0, and 2.5% (*w*/*w*). Each mixture was subjected to a thermal treatment at 80 °C for 30 min under continuous mechanical stirring at 500 rpm to ensure uniform dispersion. After heating, the samples were gradually cooled to 15 °C over 3 h, then equilibrated at 25 °C for 24 h in a low-temperature incubator. Centrifugal emulsification rates were measured as described in Section 2.2.2. The SSPS concentration that resulted in the lowest emulsification rate, indicating maximal stability, was selected for the further optimization of the heating temperature. Next, the SSPS–peanut butter mixtures were heated at temperatures ranging from 75 °C to 95 °C (in 5 °C increments) for 30 min, while all other parameters remained constant. To determine the optimal heating duration, the treatments were performed at the selected temperature for 20 to 60 min (in 10 min intervals). The duration that yielded the lowest emulsification rate was then used in the subsequent cooling experiments. Finally, the samples were cooled to different target temperatures (5, 10, 15, 20, and 25 °C) and held for 3 h under controlled conditions. The cooling temperature that provided the highest emulsification stability, again, indicated by the lowest centrifugal emulsification rate, was selected for use in the following experimental phases.

#### 2.2.4. Response Surface Methodology

Response surface methodology (RSM) was employed to optimize the processing parameters for the peanut butter with SSPS. The independent variables included SSPS concentration, heating temperature, heating time, and cooling temperature, while the centrifugal emulsification rate was used as the response variable. The experimental data were analyzed using Minitab software 21. A quadratic regression model was fitted to the data, and the model’s adequacy was evaluated through appropriate statistical tests to ensure reliability and predictive accuracy.

### 2.3. Peanut Butter Quality Determination

The peanut butter samples were prepared using the optimized processing conditions. Four types of peanut butter were produced for analysis: BSPB—peanut butter prepared from the Baisha peanut variety; SSPS-BSPB—BSPB with SSPS added; GPB—peanut butter prepared from the G965 variety; and SSPS-GPB—G965PB with SSPS added. These samples were used for subsequent quality evaluations.

#### 2.3.1. Color Analysis of Peanut Butter

Peanut butter color was assessed using a colorimeter (CM2600D, Konica Minolta (China) Investment Ltd., Shanghai, China). Twenty grams of homogenized peanut butter was evenly spread across the base of a 90 mm diameter glass Petri dish. The colorimeter was preheated and calibrated before measurement. The bottom surface of the dish was then placed against the measuring port of the colorimeter. Color readings were taken at five different points, top, bottom, left, right, and center, on the bottom surface of the dish. The average values for lightness (L*), red-green degree (a*), and yellow-blue degree (b*) were calculated and recorded to represent the overall color profile of each peanut butter sample.

#### 2.3.2. Texture Analysis

The peanut butter samples were placed into Petri dishes and subjected to texture profile analysis (TPA) and adhesiveness testing using a TA-TX2i Texture Analyzer (Stable Micro Systems Ltd, Godalming, UK). The analysis quantified key texture parameters, including hardness, cohesiveness, adhesiveness, springiness, chewiness, and gumminess. The measurement conditions were as follows: probe type—ABE35; pre-test speed—2.0 mm/s; test speed—1.0 mm/s; post-test speed—1.0 mm/s; and compression distance—10 mm.

#### 2.3.3. Nutritional Analysis

The nutritional composition of the peanut butter samples was assessed using standard analytical methods. Protein content was determined using the Kjeldahl method in accordance with Chinese National Standard GB 5009.5—2025 [24], applying a nitrogen-to-protein conversion factor of 5.46. Fat content was measured by Soxhlet extraction following GB 5009.6—2016 [25]. Sodium content was analyzed based on the method specified in GB 5009.268—2016 [26]. Energy content was calculated according to GB/Z 21922—2008 [27]. Carbohydrate content was estimated by difference, as described in GB 28050—2011 [28].

#### 2.3.4. Microstructure Analysis

The microstructure of the peanut butter samples was examined using a Hitachi S-4800 field-emission scanning electron microscope (FE-SEM; Hitachi High-Tech Corporation, Tokyo, Japan), following the method described in [29]. Prior to imaging, the samples were defatted using Soxhlet extraction with petroleum ether for at least 6 h to remove lipids. The defatted peanut butter was then freeze-dried, and the resulting powder was mounted onto a metal sample stage. A platinum coating less than 100 nm thick was applied using a vacuum ion sputtering device to enhance conductivity. The prepared samples were observed under the SEM at an accelerating voltage of 5 kV and a working distance of 9 mm to assess the microstructure of the peanut butter matrix.

### 2.4. Effect of SSPS on Shelf Life

#### 2.4.1. Sample Preparation

Peanut butter samples (500 g each) were transferred into screw-cap glass jars and stored in temperature-controlled incubators at 25 °C, 35 °C, or 45 °C to simulate accelerated shelf-life conditions. To monitor oxidative stability over time, samples (20 g aliquots) were collected at 10-day intervals for analysis from the day of production—0—to 100 days of storage. Lipid fractions were extracted from each sample using analytical-grade petroleum ether (boiling range: 30–60 °C) through solvent extraction. The resulting mixture was filtered, and the solvent was evaporated under reduced pressure (−0.1 MPa) at 50 °C to isolate the crude oil. The recovered oil was then analyzed to determine its acid value and peroxide value, which are key indicators of lipid oxidation.

#### 2.4.2. Determination of Storage Indices

The peroxide value (POV) and acid value (AV) of the extracted peanut butter oil samples were determined according to the procedures outlined in the Chinese National Standard GB/T 5009.37—2003 [30].

#### 2.4.3. Determining Kinetics of Oxidative Rancidity of Fats and Oils

The progression of oxidative rancidity in the extracted oils was modeled using first-order kinetic equations. The kinetic behavior of AV and POV was described as follows:

(2) $${Ln\left[AV\right]=At+Ln[AV}_{0}]$$

where AV_0_, AV, A, and t represent the initial acid value (mg KOH/g oil), acid value of samples (mg KOH/g oil), food rancidity rate constant (d^−1^), and storage time (d), respectively.

(3) $$Ln\left[POV\right]=Pt+Ln{[POV}_{0}]$$

where POV_0_, POV, t, and P are the initial peroxide value (g/100 g), peroxide value of samples (g/100 g), storage time (d), and food oxidation rate constant (d^−1^).

The analysis was conducted to determine the AV of peanut butter stored at various temperatures and for different durations. AVs were plotted with Ln [AV] on the y-axis and time (t) on the x-axis. The A value was obtained by fitting a linear equation to the points. The *p* value was obtained using the same method. According to the standard GB/T 16565—2003 [31], the AVs and POVs of peanut butter should not be higher than 3 mg KOH/g oil and 0.25 g/100 g, respectively.

#### 2.4.4. Determining the Shelf Life at Different Temperatures

The shelf life of peanut butter as a function of storage temperature was estimated using the following linear equation:

LnT = K S + b (4)

where S and T are shelf life (d) and storage temperature (°C), and K and b are equation constants that are derived from a linear fit. Shelf life at a specific temperature was predicted from the fitted equation.

### 2.5. Data Analysis

All experiments were conducted in triplicate, and the results were expressed as mean ± standard deviation (SD). Data processing was performed using Microsoft Excel, graphical representations were generated with Origin 8.0, and statistical analyses were carried out using Design-Expert 10.0.3. Duncan’s Multiple Range Test was applied to assess statistically significant differences among treatment groups, with a significance level set at *p* < 0.05.

## 3. Results and Discussions

### 3.1. Single-Factor Experiments

#### 3.1.1. Effect of SSPS Content on the Centrifugal Emulsification Rate

**Figure 2:**
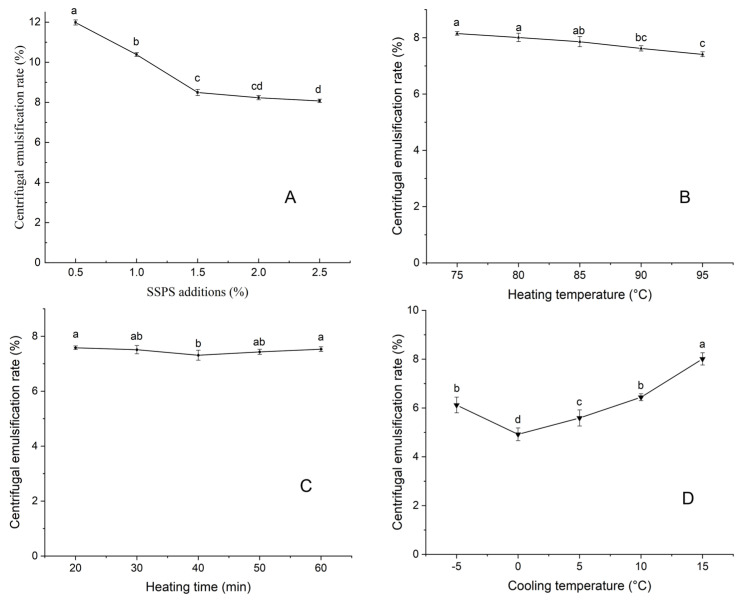
Effect of SSPS concentration (**A**), heating temperature (**B**), heating time (**C**), and cooling temperature (**D**) on the centrifugal emulsification rate of peanut butter. Different letters indicate significant differences (*p* < 0.05).

Figure 2A illustrates an inverse relationship between SSPS concentration and the centrifugal emulsification rate of SSPS-stabilized peanut butter. Specifically, the centrifugal emulsification rate decreased significantly with increasing SSPS concentration (*p* < 0.05). When the SSPS concentration exceeded 1.5%, the rate of decline in the emulsification rate became more gradual. This trend may be attributed to the anionic nature of SSPS, which allows it to engage in electrostatic interactions with peanut proteins, thereby forming a network structure [32,33]. This network effectively binds the free-flowing oil in peanut butter, restricting its migration. At an SSPS concentration of 1.5%, the centrifugal emulsification rate reached 8.49%, suggesting that the addition of SSPS in the range of 1–2% is suitable for stabilizing peanut butter.

#### 3.1.2. Effect of Heating Temperature on the Centrifugal Emulsification Rate

As shown in Figure 2B, the centrifugal emulsification rate of peanut butter decreased significantly (*p* < 0.05) with increasing heating temperature. This effect may be due to the temperature-dependent melting behavior of SSPS, which melts more completely at higher temperatures, thereby facilitating the formation of a smoother and more integrated network structure within the peanut butter matrix [34]. This enhanced structure effectively reduces oil migration, leading to a lower emulsification rate. However, at 95 °C, the peanut butter exhibited a slightly burnt taste, negatively impacting its sensory appeal and nutritional value. Considering both the emulsification performance and product quality, a heating temperature range of 80–90 °C was determined to be optimal.

#### 3.1.3. Effect of Heating Time on the Centrifugal Emulsification Rate of Peanut Butter

Overall, heating time had a relatively modest effect on the centrifugal emulsification rate. As shown in Figure 2C, a slight decrease in the centrifugal emulsification rate was observed as the heating time increased from 20 to 40 min. However, beyond 40 min, the emulsification rate began to increase again. This pattern may be attributed to the behavior of SSPS during prolonged heating. Initially, extended heating and stirring enhance the dispersion of SSPS, promoting a more complete integration of its network structure with the peanut butter matrix. This interaction helps bind oil and reduces separation. However, when heating exceeds 40 min, structural changes in the peanut proteins may occur, disrupting the already-formed SSPS network and weakening its oil-binding capacity. As a result, a heating time range of 30–50 min was considered optimal for balancing emulsification stability with nutritional quality.

#### 3.1.4. Effect of Cooling Temperature on the Centrifugal Emulsification Rate

A slight decrease in the centrifugal emulsification rate was observed as the cooling temperature increased from −5 to 0 °C (Figure 2D). This is primarily because lower cooling temperatures result in faster cooling rates, leading to the formation of smaller fat crystals. These smaller crystals contribute to a denser and more robust spatial network structure within the peanut butter, enhancing its emulsification stability [35]. However, as the cooling temperature increased from 0 °C to 15 °C, the centrifugal emulsification rate also increased, rising from approximately 5% to 8% (Figure 2D). This trend reversal suggests that slower cooling at higher temperatures leads to the formation of larger crystals and a looser network structure, which diminishes the stability of the emulsion. Therefore, a cooling temperature range of −5 to 5 °C was identified as optimal for improving emulsification stability.

### 3.2. Response Surface Optimization of Peanut Butter Processed with SSPS

#### 3.2.1. Response Surface Methodology and Analysis

To further investigate the interactive effects of the processing parameters on the centrifugal emulsification rate, RSM was applied based on the results of the single-factor experiments. A Box–Behnken design was used to assess the influence of four main factors: SSPS concentration, heating temperature, heating time, and cooling temperature. The experimental levels for each factor, along with their corresponding response values, are summarized in Table 1. Detailed experimental results and model fitting data are provided in Table 2 and Table 3, and the three-dimensional response surface plots illustrating the effects of variable interactions are shown in Figure S1.

#### 3.2.2. Developing and Validating the Response Surface Model

The experimental data were analyzed using Design-Expert 10.0.3 to derive the following regression equation for predicting the centrifugal emulsification rate:

Centrifugal emulsification rate (%) = 4.394 − 1.55167 A − 0.315 B − 0.140833 C − 0.2375 D + 0.5225 AB + 0.12 AC − 0.6175 AD − 0.755 BC − 0.4675 BD + 0.4025 CD + 2.43467 A^2^ + 1.65717 B^2^ + 1.46592 C^2^ + 1.87092 D^2^ (5)

The order of influence of the processing factors on the centrifugal emulsification rate, from most to least significant, is SSPS concentration, heating temperature, cooling temperature, and heating time (Table 3). The coefficient of determination (R^2^) for the model was 0.9734, indicating a highly significant fit. The adjusted R^2^ value was 0.9469, demonstrating that the model explains 94.69% of the variation in the response, and it closely matched the predicted R^2^, further confirming the model’s reliability and practical applicability. Based on the model, the optimal processing conditions for SSPS-stabilized peanut butter were determined to be the following: SSPS concentration of 1.663%, heating temperature of 85.344 °C, heating time of 40.355 min, and cooling temperature of 0.607 °C. Under these optimized conditions, the model predicted a centrifugal emulsification rate of 4.113%. To validate the model, peanut butter was used under the following adjusted practical conditions: 1.7% SSPS, 85 °C heating temperature, 40 min heating time, and 1 °C cooling temperature. The average centrifugal emulsification rate from three replicate experiments was 4.05%, closely aligned with the model prediction. This confirms that the response surface methodology used is both effective and feasible for optimizing SSPS-stabilized peanut butter.

### 3.3. Effect of SSPS on the Palatability of Peanut Butter

#### 3.3.1. Effect of SSPS on Color

The color parameters, namely brightness (L*), red-green (a*), and yellow-blue (b*), of four types of peanut butter are presented in Table 4. BSPB exhibited a higher L* value compared to GPB, indicating greater brightness. The incorporation of SSPS significantly reduced the L* value in both peanut butter types compared to their SSPS-free counterparts (*p* < 0.05), indicating a darker appearance. In terms of the a* value, G965-based peanut butter showed a higher redness than BS-based peanut butter. However, the addition of SSPS did not produce a statistically significant change in the a* values for either variety. For the b* values, which indicate yellowness, no significant difference was observed between the two varieties in the absence of SSPS (*p* ≥ 0.05). Nevertheless, the addition of SSPS significantly reduced the b* value of BSPB (*p* < 0.05), though it had no notable impact on the b* value of GPB (*p* ≥ 0.05). These differences in color response to SSPS addition may be attributed to the variation in particle sizes formed during processing [36]. BS peanuts, being larger, tend to undergo an incomplete Maillard reaction during roasting, producing peanut butters with a higher luminance. In contrast, the smaller G965 peanuts are more prone to a complete Maillard reaction. When combined with SSPS, this leads to a darker peanut butter, which may be considered less visually appealing.

#### 3.3.2. Effect of SSPS on Texture and Physicochemical Qualities

The hardness of both BSPB and GPB increased after the addition of SSPS, with a significant (*p* < 0.05) increase in the hardness of SSPS-GPB (Table 5). The addition of SSPS significantly (*p* < 0.05) increased the viscosity and stickiness of GPB but did not affect the viscosity and stickiness of BSPB (Table 5). The addition of SSPS simultaneously increased the adhesiveness and cohesion of both peanut butter varieties (Table 5). This may be because the pre-melted SSPS has a gel texture and contains a certain amount of water, which emulsifies with the oil in the peanut butter, thereby improving the adhesion of the peanut butter. The addition of SSPS enhanced the cohesion of the two peanut butters. The reason may be that the electrostatic interaction between the anionic groups in the SSPS structure and the amino groups in the peanut protein forms a peanut protein–polysaccharide complex. In addition, the repulsive force between the negative charges of SSPS itself makes its molecular chains stretch and disperse, making it easy to adsorb on protein particles, making the molecular network structure continuous in space, making the peanut butter texture fine [37]. The decreased viscosity in SSPS-BSPB lies in the fact that SSPS exhibits polarity, a stable structure, high solubility in water, particularly in hot water, and low viscosity [33].

The addition of SSPS did not affect protein and oil content and slightly reduced the AVs and POVs (Table 6). SSPS increases the hardness of peanut butter by forming an oil and grease network that impedes the flow of oil while also increasing the structural density of the peanut butter. The significant difference in elasticity between BSPB and GPB may be due to the different oil contents of their raw peanuts. This is supported by the fact that G965 peanuts have a higher oil content than BS peanuts, and GPB is more elastic than BSPB (*p* < 0.05). Adhesion and cohesion are also improved by SSPS, which may be due to the formation of peanut protein–polysaccharide complexes through electrostatic interactions between anionic SSPSs and the amino groups in peanut proteins [38]. Moreover, repulsive forces between negatively charged SSPS molecules can cause dispersion that facilitates adsorption with protein particles, thus enhancing the spatial continuity of the network [39].

#### 3.3.3. Effect of SSPS on the Microstructure of Peanut Butter

**Figure 3:**
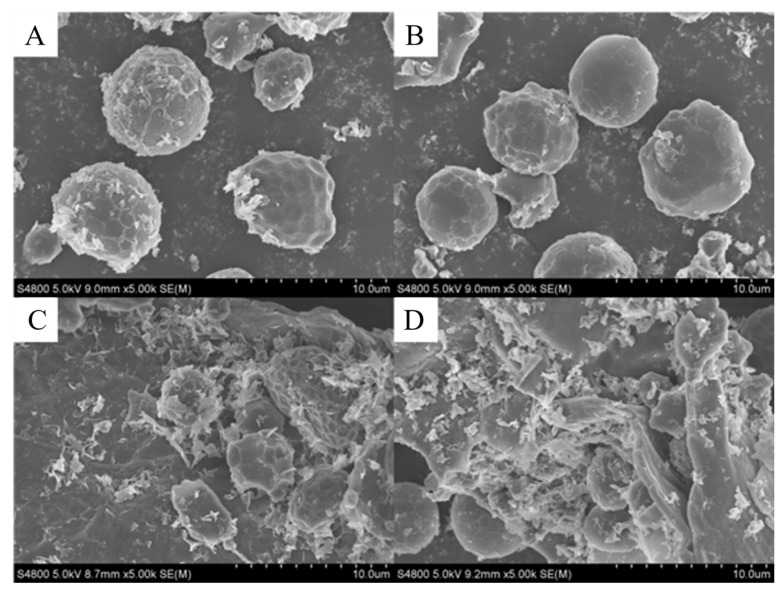
Scanning electron microscopy (SEM) of the network structure of the (**A**) BSPB, (**B**) GPB, (**C**) SSPS-BSPB, and (**D**) SSPS-GPB peanut butter varieties (BSPB: peanut butter prepared from the BS peanut variety; GPB: peanut butter prepared from the G965 variety; SSPS-BSPB: BSPB with SSPS added; SSPS-GPB: G965PB with SSPS added).

Scanning electron microscopy (SEM) was used to assess the structure of peanut butter with and without SSPS. The BSPB and GPB peanut butter varieties showed no obvious network structure to impede the flow of liquid oil and prevent oil separation (Figure 3A,B). However, the clearly visible network structure in the SSPS-BSPB and SSPS-GPB varieties demonstrated that the SSPS addition to the peanut butter allows a network to form that can impede the flow of liquid oil and prevent its separation (Figure 3C,D). This may be due to the electrostatic interactions between the peanut protein and SSPS in the peanut butter [23]. The formed network structure has a certain oil-binding capacity, hindering the oil flow in the sauce and reducing the centrifugal creaming rate of the peanut butter.

### 3.4. Effect of SSPS on the Shelf Life of Peanut Butter

#### 3.4.1. AV and POV at Different Storage Temperatures and Times

**Figure 4:**
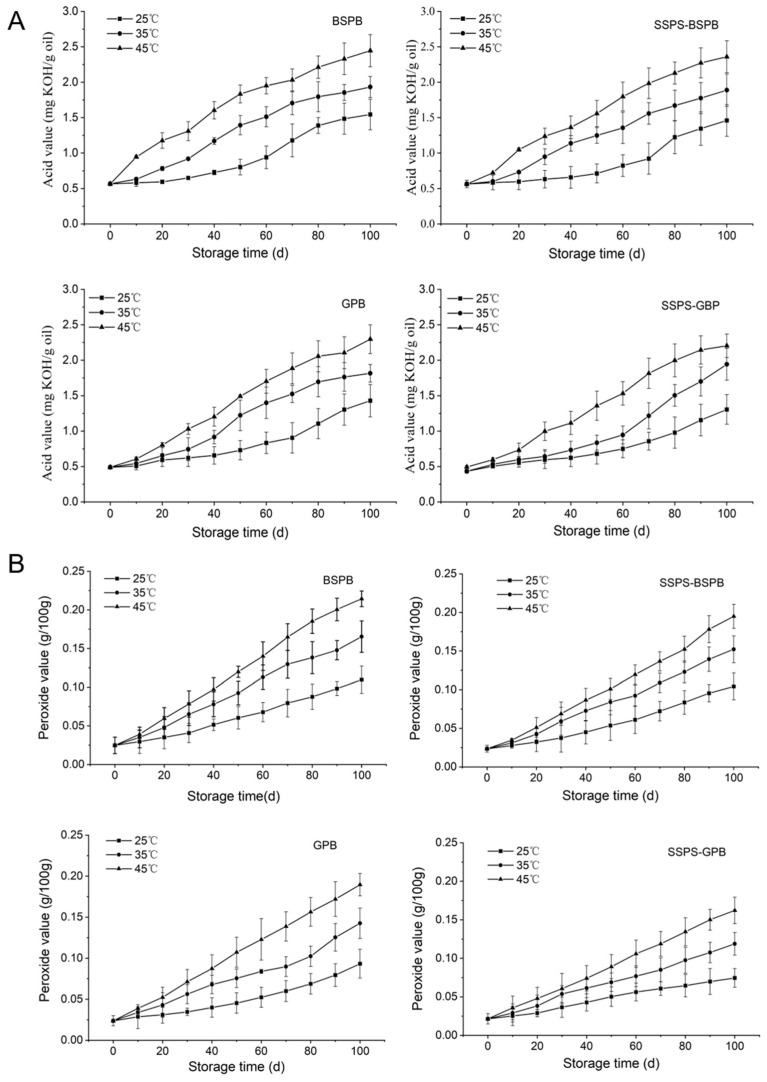
The relationship between the acid value (**A**) and peroxide value (**B**) of peanut butter over storage time (BSPB: peanut butter prepared from the BS peanut variety; SSPS-BSPB: BSPB with SSPS added; GPB: peanut butter prepared from the G965 variety; SSPS-GPB: G965PB with SSPS added).

The AVs and POVs of BSPB, SSPS-BSPB, GPB, and SSPS-GPB did not exceed the standard during the 100-day storage period. There was a positive correlation between the storage time and AV at all storage temperatures for all four peanut butter types (Figure 4A). There was also a positive correlation between the storage temperature and AV at all storage times for all four peanut butter types. The peanut butter with added SSPS generally had a lower AV at most storage times and temperatures compared to BSPB and GPB. Additionally, the AV of GPB was lower than that of BSPB for most storage times and temperatures. Similarly, the general trends for the POVs were largely consistent with those observed in the AVs (Figure 4B). The initial AV of the peanut butter with SSPS added was lower than that without SSPS, and the subsequent increase in storage was also lower than that of the peanut butter without SSPS. This may be because SSPS has a certain antioxidant property [20], which can inhibit the increase in AV in peanut butter. In addition, the network structure formed by SSPS in peanut butter can hinder the flow of oil in the butter, reduce the centrifugal emulsion rate of the peanut butter, and further reduce the occurrence of peanut butter rancidity. A comparison of the data at the same temperature in Figure 4A shows that the initial AV of the G965 peanut butter was lower than that of the BS-variety peanut butter. This is because the proportion of oleic acid in G965 peanuts is high, and oleic acid itself has good stability and does not easily undergo oxidative rancidity reactions [40]. The above results show that the addition of SSPS is beneficial to enhance the oil stability of peanut butter and inhibit increases in the AV and POV of peanut butter so that the peanut butter can maintain a higher quality for a longer period of time.

#### 3.4.2. Kinetic Analysis of Oxidative Rancidity

Linear kinetic equations were developed to explain the variations in AV and POV in each of the four types of peanut butter as they were kept at temperatures of 25, 35, and 45 °C. The equations outlined in Table 7 and Table 8 demonstrated a strong correlation (R^2^ > 0.9). According to the Chinese National Standard GB/T 16565—2003 [31], the maximum AV and POV allowed for peanut butter is 3 mg KOH/g oil and 0.25 g/100 g, respectively. The equations utilize the highest allowable AV and POV values to forecast the shelf life of each variety of peanut butter at different storage temperatures. Temperature-dependent shelf life predictions revealed progressive decreases with increasing storage temperatures. BSPB demonstrated shelf lives of 155 d (25 °C), 120 d (35 °C), and 100 d (45 °C). SSPS incorporation extended these values to 178 d, 127 d, and 105 d, respectively, for SSPS-BSPB. Similar enhancements were observed in the GPB formulations, with control GPB lasting 173 d, 123 d, and 105 d versus SSPS-GPB’s 194 d, 133 d, and 108 d at the corresponding temperatures. When using POV to predict shelf life, BSPB lasted 149, 112, and 95 days, respectively. SSPS-BSPB lasted 153, 117, and 102 days. GPB lasted 179, 131, and 104 days, and SSPS-GPB lasted 185, 138, and 112 days, respectively.

**Figure 5:**
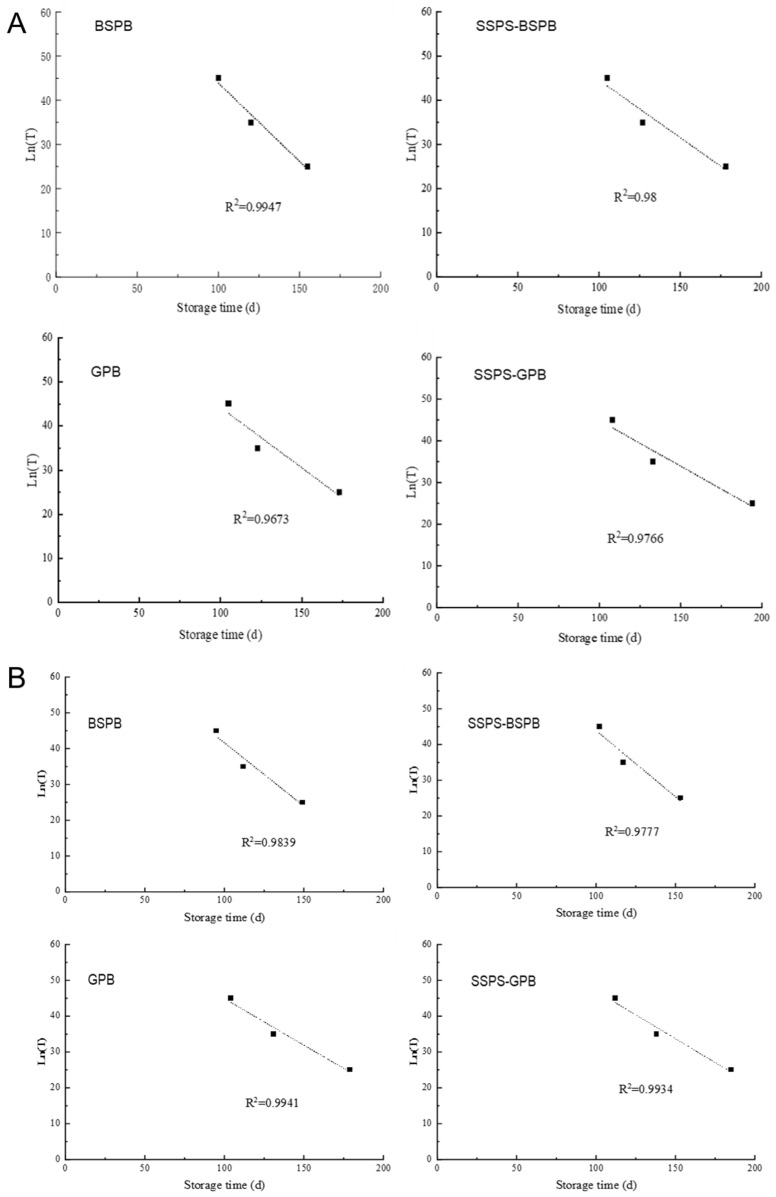
Acid values (**A**) and peroxide values (**B**) of peanut butter at different storage temperatures (BSPB: peanut butter prepared from the BS peanut variety; SSPS-BSPB: BSPB with SSPS added; GPB: peanut butter prepared from the G965 variety; SSPS-GPB: G965PB with SSPS added).

The shelf life equations for the four peanut butter types at various storage temperatures were analyzed and fitted across different temperatures and time points, as shown in Figure 5A,B.

#### 3.4.3. Shelf Life Validation Test

The shelf life of each peanut butter variety at each storage temperature was experimentally determined and compared with the model predictions. The shelf life predictions had a relative error below 5% (Table 9), demonstrating that the shelf life of the four peanut butter varieties could be accurately predicted at each of these storage temperatures quickly and reliably using these models. The use of POVs in the kinetic models resulted in an average relative error of 2.83% between the predicted and experimental shelf lives. This was smaller than the average relative error when the acid value was used, which was 3.06%. Additionally, the use of POVs resulted in a better model fit. These results, shown in Table 9, indicate that the use of POVs leads to a more accurate model. Both the predicted and experimental shelf life results showed that the addition of SSPS significantly increased the shelf life of peanut butter.

## 4. Conclusions

This study established the following optimal processing parameters for peanut butter: 1.7% SSPS concentration, heating at 85 °C for 40 min, and cooling to 1 °C. Under these optimal conditions, the centrifugal emulsification rate (4.05%) closely matched the predicted value (4.11%). Comparative analysis revealed that blanched-skin peanut butter (BSPB) exhibited a higher L* value than germinated peanut butter (GPB) (*p* < 0.05). SSPS incorporation significantly reduced L* values in both varieties (*p* < 0.05). SSPS addition also increased the cohesiveness, hardness, moisture content, ash content, and carbohydrate content of the peanut butter, while slightly reducing AVs and POVs. The developed SSPS-stabilized peanut butter demonstrates commercial potential due to its extended shelf stability. Furthermore, SSPS stabilization improved the shelf life and enhanced the nutritional quality of peanut butter. Moreover, utilizing SSPS promotes the high-value use of soybean by-products and supports the development of innovative peanut-based products. Peanut butter has a positive effect on multiple qualities. In summary, the findings demonstrate that SSPS addition positively affects multiple quality attributes of peanut butter.

## Figures and Tables

**Table 1:** Response surface methodology results.

Code	SSPS Content A/(%)	Heating Temperature B/(°C)	Heating Time C/(min)	Cooling Temperature D/(°C)
−1	1.0	80	30	−5
0	1.5	85	40	0
1	2.0	90	50	5

**Table 2:** Experimental design and results.

No.	SSPS Content A/(%)	Heating Temperature B/(°C)	Heating Time C/(min)	Cooling Temperature D/(°C)	Centrifugal Emulsification Rate (%)
1	0	−1	1	0	8.58
2	0	1	−1	0	8.09
3	1	0	0	−1	8.45
4	1	1	0	0	7.42
5	0	1	0	1	7.09
6	−1	1	0	0	8.91
7	−1	0	0	1	10.31
8	−1	0	0	−1	10.15
9	1	0	0	1	6.14
10	0	0	1	1	8.13
11	1	0	1	0	6.05
12	0	−1	−1	0	7.36
13	0	0	−1	1	7.43
14	0	0	0	0	4.22
15	0	0	0	0	4.11
16	0	0	0	0	4.41
17	0	−1	0	1	8.18
18	−1	0	1	0	9.65
19	0	0	0	0	4.51
20	0	1	1	0	6.29
21	0	−1	0	−1	7.38
22	0	0	0	0	4.72
23	1	0	−1	0	6.26
24	−1	−1	0	0	10.91
25	1	−1	0	0	7.33
26	0	0	1	−1	7.54
27	−1	0	−1	0	10.34
28	0	1	0	−1	8.16
29	0	0	−1	−1	8.45

**Table 3:** ANOVA results of the fitted regression equation for the response surface centrifugal emulsification rate.

Source of Variance	Sum of Squares	Degrees of Freedom	Variance	F-Value	*p*-Value	Significance
Regression model	98.78	14	7.06	36.65	<0.0001	**
A	28.89	1	28.89	150.07	<0.0001	**
B	1.19	1	1.19	6.18	0.0261	*
C	0.24	1	0.24	1.24	0.2849	
D	0.68	1	0.68	3.52	0.0818	
AB	1.09	1	1.09	5.67	0.0320	*
AC	0.058	1	0.058	0.30	0.5930	
AD	1.53	1	1.53	7.92	0.0138	*
BC	2.28	1	2.28	11.84	0.0040	**
BD	0.87	1	0.87	4.54	0.0513	
CD	0.65	1	0.65	3.37	0.0879	
A2	38.45	1	38.45	199.72	<0.0001	**
B2	17.81	1	17.81	92.53	<0.0001	**
C2	13.94	1	13.94	72.40	<0.0001	**
D2	22.70	1	22.70	117.94	<0.0001	**
Residual	2.70	14	0.19			
Lost proposal	2.46	10	0.25	4.27	0.0873	
Pure error	0.23	4	0.058			
Total	101.47	28				

R^2^ = 0.9734; Adj R^2^ = 0.9469; Pred R^2^ = 0.8566; * significant effect on the results (*p* < 0.05); ** highly significant effect on the results (*p* < 0.01).

**Table 4:** Chromaticity values.

Sample Name	L*	a*	b*
BSPB	63.99 ± 0.30 ^a^	10.79 ± 0.20 ^b^	32.01 ± 2.06 ^a^
SSPS-BSPB	58.93 ± 1.91 ^b^	10.37 ± 0.44 ^b^	28.77 ± 1.01 ^b^
GPB	58.81 ± 0.09 ^b^	13.74 ± 0.03 ^a^	32.23 ± 0.21 ^a^
SSPS-GPB	55.91 ± 0.33 ^c^	13.97 ± 0.12 ^a^	31.70 ± 0.09 ^a^

BSPB: Peanut butter prepared from the BS peanut variety; SSPS-BSPB: BSPB with SSPS added; GPB: peanut butter prepared from the G965 variety; SSPS-GPB: G965PB with SSPS added. Brightness (L*), red-green (a*), and yellow-blue (b*) values were quantified for each type of peanut butter. Different letters indicate significant differences (*p* < 0.05).

**Table 5:** Texture analysis.

Indexes	Sample Names			
	BSPB	SSPS-BSPB	GPB	SSPS-GPB
Hardness (g)	58.33 ± 6.65 ^ab^	67.12 ± 6.08 ^a^	48.56 ± 6.92 ^c^	58.41 ± 14.42 ^ab^
Viscosity (g)	21.44 ± 3.61 ^a^	20.79 ± 0.00 ^a^	23.18 ± 1.00 ^ab^	38.67 ± 35.81 ^b^
Gumminess (mJ)	4.55 ± 1.05 ^ab^	4.95 ± 0.70 ^ab^	2.74 ± 0.95 ^c^	4.37 ± 0.21 ^a^
Elasticity	0.04 ± 0.01 ^a^	0.04 ± 0.01 ^a^	0.05 ± 0.01 ^a^	0.04 ± 0.01 ^a^
Springiness (mm)	6.03 ± 0.05 ^ab^	6.42 ± 0.18 ^a^	5.64 ± 0.18 ^bc^	5.23 ± 0.71 ^c^
Elastic length (mm)	0.93 ± 0.11 ^b^	6.67 ± 0.54 ^a^	5.65 ± 4.24 ^ab^	4.71 ± 3.25 ^ab^
Cohesion	1.12 ± 0.02 ^ab^	1.27 ± 0.02 ^a^	1.09 ± 0.01 ^ab^	1.31 ± 0.12 ^ab^
Adhesiveness (g)	70.66 ± 8.72 ^ab^	74.33 ± 7.37 ^a^	52.67 ± 7.23 ^c^	60.67 ± 16.62 ^ab^
Chewiness (mJ)	4.07 ± 0.42 ^a^	4.37 ± 0.56 ^a^	3.09 ± 0.36 ^ab^	3.13 ± 1.06 ^ab^

BSPB: Peanut butter prepared from the BS peanut variety; SSPS-BSPB: BSPB with SSPS added; GPB: peanut butter prepared from the G965 variety; SSPS-GPB: G965PB with SSPS added. Different letters indicate significant differences (*p* < 0.05).

**Table 6:** Nutritional and physicochemical analysis.

Indexes	Standard Content	Sample Names			
		BSPB	SSPS-BSPB	GPB	SSPS-GPB
Moisture (%)	≤1.0	0.72	0.83	0.69	0.79
Ash (%)	≤3.0	2.4	2.5	2.4	2.6
Protein (%)	≥23.0	28.3	27.8	23.0	22.2
Fat (%)	≥40.0	50.9	48.2	48.8	51.1
Carbohydrate (%)		15.8	17.6	22.8	20.3
Sodium (mg/100 g)		276	331	273	316
Energy (kJ)		2633	2555	2584	2613
Acid value (mg KOH/g oil)	≤3.0	0.573	0.561	0.482	0.475
Peroxide value (g/100 g)	≤0.25	0.024	0.023	0.023	0.021

BSPB: Peanut butter prepared from the BS peanut variety; SSPS-BSPB: BSPB with SSPS added; GPB: peanut butter prepared from the G965 variety; SSPS-GPB: G965PB with SSPS added.

**Table 7:** Regression analysis of the acid value of peanut butter processed at different storage temperatures for a fixed period.

	Temperature (°C)	Regression Equation	R^2^	Formula
BSPB	25	Ln(AV) = 0.0116 t − 0.7036	0.9585	6
	35	Ln(AV) = 0.0131 t − 0.4798	0.9426	7
	45	Ln(AV) = 0.0141 t − 0.3236	0.9058	9
SSPS-BSPB	25	Ln(AV) = 0.0102 t − 0.7155	0.9214	10
	35	Ln(AV) = 0.0127 t − 0.5125	0.9565	11
	45	Ln(AV) = 0.0137 t − 0.3398	0.9228	13
GPB	25	Ln(AV) = 0.0109 t − 0.7907	0.9746	14
	35	Ln(AV) = 0.0145 t − 0.6806	0.9589	15
	45	Ln(AV) = 0.0156 t − 0.5382	0.9416	17
SSPB-GPB	25	Ln(AV) = 0.0098 t − 0.8037	0.9655	18
	35	Ln(AV) = 0.0144 t − 0.8153	0.9789	19
	45	Ln(AV) = 0.0156 t − 0.5843	0.9618	20

BSPB: Peanut butter prepared from the BS peanut variety; SSPS-BSPB: BSPB with SSPS added; GPB: peanut butter prepared from the G965 variety; SSPS-GPB: G965PB with SSPS added. AV: Acid value.

**Table 8:** Regression analysis of the peroxide value of peanut butter processed at different storage temperatures for a fixed period.

	Temperature (°C)	Regression Equation	R^2^	Formula
BSPB	25	Ln(POV) = 0.0151 t − 3.6374	0.9889	21
	35	Ln(POV) = 0.0183 t − 3.4307	0.9401	22
	45	Ln(POV) = 0.0205 t − 3.3316	0.9276	23
SSPS-BSPB	25	Ln(POV) = 0.0153 t − 3.7275	0.9970	25
	35	Ln(POV) = 0.0182 t − 3.5301	0.9525	26
	45	Ln(POV) = 0.0201 t − 3.4438	0.9440	27
GPB	25	Ln(POV) = 0.013 t − 3.7137	0.9947	29
	35	Ln(POV) = 0.016 t − 3.4834	0.9655	30
	45	Ln(POV) = 0.019 t − 3.3610	0.9410	31
SSPS-GPB	25	Ln(POV) = 0.0128 t − 3.7482	0.9610	33
	35	Ln(POV) = 0.0161 t − 3.6031	0.9413	34
	45	Ln(POV) = 0.0188 t − 3.4984	0.9393	35

BSPB: Peanut butter prepared from the BS peanut variety; SSPS-BSPB: BSPB with SSPS added; GPB: peanut butter prepared from the G965 variety; SSPS-GPB: G965PB with SSPS added. POV: Peroxide value.

**Table 9:** Comparison of the predicted and actual shelf life of peanut butter upon SSPS addition.

Sample Type	Storage Temperature (°C)	Acid Value Model			Peroxide Value Model		
		Shelf Life Prediction (d)	Shelf Life Actual (d)	Relative Error (%)	Shelf Life Prediction (d)	Shelf Life Actual (d)	Relative Error (%)
BSPB	20	177.2	170	4.23	168.7	163	3.50
SSPS-BSPB	20	204.7	200	2.35	172.1	169	1.83
GPB	20	198.9	194	2.52	207.7	203	1.97
SSPS-GPB	20	225.2	219	2.73	213.0	207	2.89

Relative error = (predicted value − measured value)/measured value × 100% (BSPB: peanut butter prepared from the BS peanut variety; SSPS-BSPB: BSPB with SSPS added; GPB: peanut butter prepared from the G965 variety; SSPS-GPB: G965PB with SSPS added).

## Data Availability

The original contributions presented in this study are included in the article. Further inquiries can be directed to the corresponding author.

## Supplementary Materials

The following supporting information can be downloaded at https://www.mdpi.com/article/10.3390/foods14132180/s1, Figure S1: Response surface methodology and contour plots illustrating the interactive effects of processing parameters on the centrifugal emulsification rate of peanut butter (A: SSPS concentration vs. heating temperature; B: SSPS concentration vs. heating time; C: SSPS concentration vs. cooling temperature; D: Heating temperature vs. heating time; E: Heating temperature vs. cooling temperature; and F: Heating time vs. cooling temperature).
