# Supplementary material for: Soluble Soybean Polysaccharide Improves Quality and Shelf Life of Peanut Butter
Source: Foods. 2025 Jun 22;14(13):2180. doi: 10.3390/foods14132180 (PMC12248660; doi:10.3390/foods14132180)
Supplement: Supplementary file 1 [file foods-14-02180-s001.zip › foods-3682001-supplementary.pdf]

Soluble Soybean Polysaccharide Improves the Quality and Shelf Life of Peanut Butter

Liangchen Zhang [1,†], Liyou Zheng [2,†], Jian Sun [3], Sameh A. Korma [4,5], Fahad Al-Asmari [6],

Mengxi Xie [1], and Miao Yu [1,*]

1 Institute of Food and Processing, Liaoning Academy of Agricultural Sciences, Shenyang, 110161, China; napoleon19831214@163.com (L.C.Z.), moor1112@163.com (M.X.), jannytiti@163.com (M.Y.)

2 School of Biological and Food Engineering, Anhui Polytechnic University, Wuhu, 241000, China; zhengliyou@ahpu.edu.cn (L.Y.Z.)

3 Department of Food Science, Shenyang Agricultural University, Shenyang, 110866, China; 15020127746@163.com (J.S.)

4 Department of Food Science, Faculty of Agriculture, Zagazig University, Zagazig, 44519, Egypt; sameh.hosny@zu.edu.eg (S.A.K.)

5 School of Food Science and Engineering, South China University of Technology, Guangzhou, 510641, China

6 Department of Food Science and Nutrition, College of Agriculture and Food Sciences, King Faisal Uni-versity, P.O. Box 400, Al-Ahsa, 31982 Al-Hofuf, Saudi Arabia; falasmari@kfu.edu.sa (F.A.A)

† These authors contributed equally to this work and shared first authorship.

* Correspondence: jannytiti@163.com (M.Y.); Tel.: +86 159 – 9837 – 8968 (M.Y.)2.3 Oil extraction

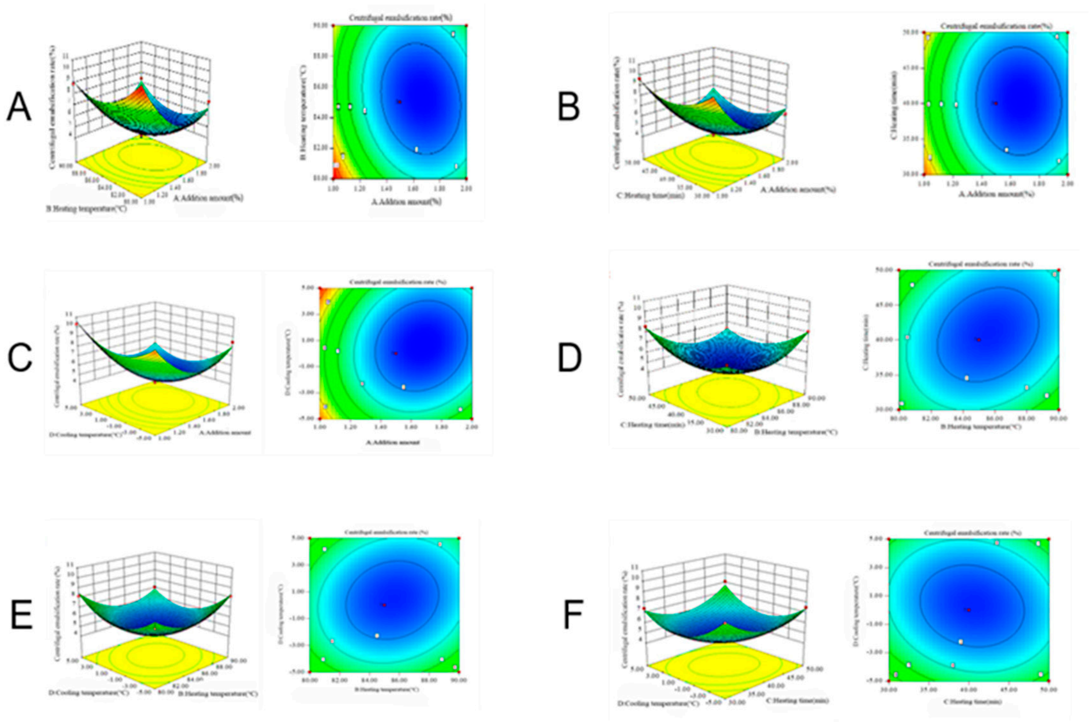

Figure S1. Response surface methodology and contour plots illustrating the interactive effects of processing parameters on the centrifugal emulsification rate of peanut butter (**A**: SSPS concentration vs. heating temperature; **B**: SSPS concentration vs. heating time; **C**: SSPS concentration vs. cooling temperature; **D**: Heating temperature vs. heating time; **E**: Heating temperature vs. cooling temperature; and **F**: Heating time vs. cooling temperature).
